# Unveiling the Web: Exploring the Multifaceted Role of Neutrophil Extracellular Traps in Ocular Health and Disease

**DOI:** 10.3390/jcm13020512

**Published:** 2024-01-17

**Authors:** Salma Adeeb, Tarek Ziad Arabi, Hassan Shah, Sulaiman Alsalameh, Mylia Abu-Shaar, Abduljalil Mohamed El-Sibai, Khaled Alkattan, Ahmed Yaqinuddin

**Affiliations:** College of Medicine, Alfaisal University, Riyadh 11533, Saudi Arabia; snadeeb@alfaisal.edu (S.A.); tarabi@alfaisal.edu (T.Z.A.); hshah@alfaisal.edu (H.S.); salsalameh@alfaisal.edu (S.A.); mshaar@alfaisal.edu (M.A.-S.); aelsibai@alfaisal.edu (A.M.E.-S.); kkattan@alfaisal.edu (K.A.)

**Keywords:** neutrophils, neutrophil extracellular traps, uveitis, diabetic retinopathy, corneal diseases

## Abstract

Neutrophil extracellular traps (NETs) play an essential role in antimicrobial defense. However, NETs have also been shown to promote and mediate a wide spectrum of diseases, including cancer, diabetes mellitus, cardiovascular diseases, and ocular diseases. Data regarding NETs in ocular diseases remain limited. In physiological conditions, NETs protect the eye from debris and cleave proinflammatory cytokines, including several interleukins. On the other hand, NETs play a role in corneal diseases, such as dry eye disease and ocular graft-versus-host disease, where they promote acinar atrophy and delayed wound healing. Additionally, NET levels positively correlate with increased severity of uveitis. NETs have also been described in the context of diabetic retinopathy. Although increased NET biomarkers are associated with an increased risk of the disease, NETs also assist in the elimination of pathological blood vessels and the regeneration of normal vessels. Targeting NET pathways for the treatment of ocular diseases has shown promising outcomes; however, more studies are still needed in this regard. In this article, we summarize the literature on the protective roles of NETs in the eye. Then, we describe their pathogenetic effects in ocular diseases, including those of the cornea, uvea, and retinal blood vessels. Finally, we describe the therapeutic implications of targeting NETs in such conditions.

## 1. Introduction

Neutrophils constitute the largest portion of circulating leukocytes and represent the first line of defense against pathogenic germs. In addition to traditional antimicrobial processes, unique web-like structures, termed neutrophil extracellular traps (NETs), which are formed and released by highly active neutrophils, were discovered [1]. NETs consist of extracellular deoxyribonucleic acid (eDNA) filaments coated with histones and neutrophil granule proteins [2]. Myeloperoxidase (MPO), neutrophil elastase (NE), lactoferrin, cathepsins, calprotectin, LL-37, and defensin are among several proteins identified and discovered in NETs [2,3,4].

Neutrophils and NETs are important components of the immune system and overall health. NETs play a critical role in host defense, especially when neutrophilic functions are overwhelmed by invading microbes. Specifically, NETs prevent the growth and spread of several bacteria, including *Staphylococcus* aureus, *Veillonella parvula*, and *Streptococcus gordonii* [5,6]. NETs also perform antifungal activities against *Candida albicans*, *Aspergillus fumigatus*, and *Trichophyton rubrum*, among others [7,8].

However, mounting evidence suggests that NETs have a role in the etiology of a variety of disorders, including diabetes, cardiovascular disease, and autoimmune disease [9,10,11]. For example, in diabetes, NETs are implicated in exacerbating inflammation and tissue damage, which are critical in the progression of vascular complications. The formation of NETs in diabetic conditions is associated with endothelial dysfunction and contributes to the pathogenesis of diabetic complications, including nephropathy [12].

In the context of cardiovascular diseases, NETs play a crucial role in atherogenesis and thrombosis [13]. They are involved in promoting endothelial dysfunction, smooth muscle cell proliferation, and arterial lipid accumulation, leading to atherosclerotic plaque formation [13]. Additionally, NETs facilitate thrombotic events, further complicating cardiovascular conditions [13].

As a result, NETs have piqued the curiosity and attention of specialists from a wide range of fields. This has further expanded into the field of ophthalmology, where researchers discovered that NETs have opposing functions in the ocular system for neutrophils in normal and pathological environments. For example, NETs play a pathological role in uveitis, dry eye disease, and many other pathologies [14,15,16]. In this review, we aim to summarize the latest literature on the role of NETs in physiological and pathological conditions. Then, we describe the implications of targeting NETs in the treatment of several ocular pathologies.

## 2. Mechanism of NET Formation

NETs are classically formed through a process called NETosis, which leads to the release of NETs into extracellular spaces and the death of the activated neutrophil [17]. However, other pathways that avoid neutrophil death have also been described [18].

**Figure 1:**
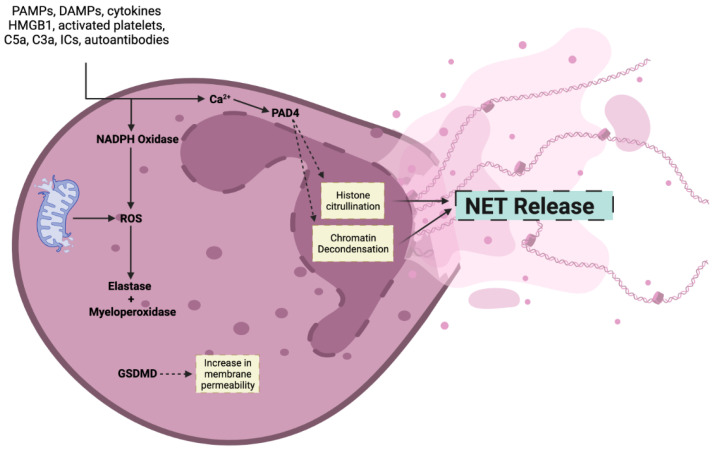
NET formation is triggered by several external stimuli, which increase intracellular calcium. Subsequently, histone citrullination and chromatin decondensation occur, causing NET release.

NET ejection from neutrophils can be induced by a variety of stimuli, including invasive pathogens, bacterial lipopolysaccharides (LPS), abnormal conditions in vivo (e.g., high bicarbonate and hyperglycemia), and chemical structures in vitro (e.g., phorbol 12-myristate 13-acetate (PMA) and sodium hydroxide) (Figure 1) [19]. To date, the creation of NETs in response to various stimuli is not fully understood. Nonetheless, there are two commonly acknowledged mechanisms for NET generation: the NADPH oxidase (Nox)-dependent pathway and the Nox-independent pathway, with the latter being a more novel and less studied pathway [2,20].

The suicidal NETosis model has been described as dependent on protein kinase C activity (PKC) as the major regulator of this pathway, which is also Nox-2 dependent. The Nox-dependent pathway begins with the identification of any stimulus that activates receptors, which causes signal-related kinases such as PKC, p38, PI3K, Src, Raf/MERK/Erk, Akt, and Ark to be activated [21,22]. These pathways eventually lead to increased cytosolic calcium levels, which in turn activate Nox. Further downstream, the Nox multimeric complex assembles at the phagosomal membrane and creates reactive oxidative species (ROS). ROS production promotes protease neutrophil elastase (NE) and myeloperoxidase (MPO) activation, which digest the nuclear membrane and decondense nuclear chromatin. MPO and NE are key components of NETs and play pivotal roles in their function and structure. MPO, a heme protein primarily stored in the azurophilic granules of neutrophils, contributes significantly to microbial killing within NETs. It catalyzes the production of hypochlorous acid from hydrogen peroxide and chloride ions, a reaction central to the antimicrobial properties of NETs [23]. MPO also modulates the formation of NETs by regulating key signaling pathways and is involved in the complex interplay of immune responses within the NET structure [23].

NE, another granule protein, is crucial for degrading extracellular matrix components and facilitating the NET formation process [17]. NE translocates to the nucleus of the neutrophil, where it partially degrades histones, leading to chromatin decondensation, a key step in NETosis. This process liberates DNA and associated proteins into the extracellular space, forming NETs. NE’s activity within NETs extends to modulating inflammation and the immune response, particularly in diseases where NET formation is dysregulated [24,25].

Cytosolic calcium also activates peptidylarginase deaminase-4 (PAD4), an enzyme that further promotes histone deamination and thereby chromatin decondensation [26]. When the nuclear membrane ruptures, granular proteins and DNA combine to create NETs, which are ejected into the extracellular compartment as the cell membrane disintegrates, eventually causing neutrophil death [2].

Neutrophils from patients with chronic granulomatous disease, a primary immunodeficiency caused by mutations in Nox subunits, demonstrate blunted NET production in response to mitogens and certain microorganisms. However, some NET triggers, including immune complexes, ionomycin, and nicotine, have been postulated to cause NET release independently of Nox, relying instead on mitochondrial ROS [2,4].

However, not all NET formation leads to neutrophil death. Another form of NET release, known as non-lytic NETosis, occurs through a Nox-independent route and does not require ROS generation or result in cell death. Bacteria that activate toll-like receptors (TLRs) and complement-mediated mechanisms, such as *S. aureus*, might cause non-lytic NETosis. In contrast to lytic NETosis, the antimicrobial effects of neutrophils, such as chemotaxis and phagocytosis degranulation, are conserved because plasma membranes stay intact and active phagolysosomes are reserved [27].

Not all stimuli activate the main proteins listed above. Guiducci et al., for example, revealed that PAD4 was not required for the process of Candida albicans-induced NETosis [28]. Since PAD4 inhibitors are commonly used to target NETs [29,30], non-PAD4-dependent NET formation could render patients resistant to treatment in certain conditions.

The contents of NETs vary largely as well. For example, LPS-induced NETs and spontaneous NETs are similar in protein content and post-translational modifications, but different from PMA-induced NETs [31]. Additionally, different NET components, such as methyl-oxidized α-enolase and sulphoxide methionine 93, can be found at elevated levels in systemic lupus erythematosus and lupus nephritis compared with controls [32]. Sulphoxide methionine constitutes part of the peptide that is targeted by autoantibodies of lupus nephritis patients [32]. *P. aeruginosa* induces LL37-dense NET formation, which has been shown to have greater antibacterial effects against the bacteria [33]. Collectively, these findings demonstrate that the constituents of NETs vary according to the stimuli and environment, which adds to their adaptive role physiologically and pathologically.

## 3. NETs in Ocular Homeostasis

The first leukocytes to be attracted to different tissues during inflammation are neutrophils. In the absence of ocular disease or infection, a small number of neutrophils are recruited, activated, and penetrate the tear film to maintain ocular homeostasis. In tear fluid, activated complements, such as C5a and neutrophil chemoattractants, aid in neutrophil recruitment [34]. The blinking activity of the eyes promotes continual tear replenishment and increases neutrophil infiltration and degranulation, especially in closed eyes [35,36]. During eye closure, these coordinated and intricate activities are accompanied by spontaneous NET creation [34].

The ocular rheum is exposed to the environment, which may include dust, particulates, bacteria, LPS, and antimicrobial molecules such as IgA, complements, and lactoferrin. When compared with closed eyelids, the environment of the ocular surface of the open eye evolves toward a condition with less CO_2_ and more O_2_. This mild alkalinization promotes NETosis, which is also concurrently exacerbated by LPS and bacteria. These foreign objects and detritus are enwrapped by the NETs in the conjunctival area, and the exudates are released from the ocular surface via the canthi upon eye closure [34]. The finding of NETs circulating in the ocular rheum reinforces neutrophils’ prominent function under physiological settings.

Furthermore, the serine proteases in NETs are responsible for the characteristic proteolytic effects of ocular rheum of inflammatory mediators such as interleukin (IL)-12 and IL-17A and monocyte chemoattractant protein-1 [34]. Conclusively, NETs play an important role in blunting ocular inflammation and host defense against microbes and debris.

## 4. NETs in Bacterial Infections of the Eye

Neutrophils play a crucial role in the ocular defense against pathogenic bacteria, including *S. aureus*, *Chlamydia caviae*, and *Pseudomonas aeruginosa* [16,37,38]. *P. aeruginosa* is a leading cause of bacterial keratitis due to the resistant biofilms it produces and its type-3 secretion system (T3SS), which releases toxins into host cells [39]. The role of NETs in the defense against ocular *P. aeruginosa* infections is relatively unknown. Thanabalasuriar et al. analyzed the role of neutrophils and NETs in *P. aeruginosa* keratitis mice models [38]. First, the authors demonstrated that neutrophils are unable to penetrate the *P. aeruginosa*-produced biofilms. The NET release was triggered by the detection of T3SS. Subsequently, NETs formed a “dead-zone” barrier that surrounds the bacteria and limits it to the external ocular environment. Although NETs prevent bacterial spread to the brain, confinement of *P. aeruginosa* led to greater antibiotic resistance [38]. Zhu et al. also set out to investigate the role of NETs in *P. aeruginosa* keratitis [40]. The authors revealed that despite NETs enhancing *P. aeruginosa* clearance, they cause concurrent corneal damage. Furthermore, dexamethasone and tobramycin blunted NET production, indicating their potential for targeting NETs.

Equine recurrent uveitis (ERU), a common disease among horses, is considered an accurate model for human autoimmune uveitis. *Leptospira* bacteria are considered a risk factor for the development of ERU [15]. Fingerhut et al. assessed the role of NETs in ERU and the association between NET markers and disease severity [15]. ERU-diseased horses had significantly greater NET-related markers (i.e., cell-free DNA and DNase-1) in serum and vitreous body fluids. Additionally, the levels of several NET markers in the vitreous body fluid correlated positively with disease severity. Similarly, vitreous body fluid NET marker levels were positively associated with the presence of *Leptospira* bacteria. NETs demonstrated significant cytotoxic effects on the blood–retina barrier. Direct data reporting the role of NETs in fending off other bacterial infections, such as *S. aureus*, in the eye are not yet available to the best of our knowledge.

## 5. NETs in Fungal Infections of the Eye

Fungi constitute approximately half of the keratitis cases in the developing world [41]. Most frequently caused by the *Aspergillus* and *Fusarium* species, these cases are very complex due to their refractory nature and delayed diagnosis and treatment [41]. In fungal keratitis, mast cells protect corneal tissue through vasodilation, enhancing neutrophil recruitment [42]. Furthermore, the activation of TLRs -4 and -5 following corneal injury promotes chemoattractant release [43].

Jin et al. assessed the presence of NETs in fungal keratitis [44]. Their study revealed that NETs were present in all 14 patients included in the analysis. Greater NET formation was associated with a better treatment response and shorter duration of the disease. Then, Fan et al. demonstrated that corticosteroid treatment exacerbates *C. albicans* keratitis by inhibiting NET formation and neutrophil infiltration in mice models [45]. Furthermore, NETs have been found in around three-quarters of COVID-19-associated rhino-orbital-cerebral mucormycosis. Collectively, these findings demonstrate that NETs are crucial for antifungal host defense and that inhibiting NET formation predisposes to more severe disease.

## 6. NETs in Protozoal Infections of the Eye

*Acanthamoeba* keratitis is a rare, sight-threatening protozoal infection caused by *Acanthamoeba* species [46,47]. Evidence has suggested that proteases are utilized by *Acanthamoeba* to mediate protozoal infection, promote inflammatory cell apoptosis, and destroy keratocytes, endothelial cells, and ciliary body cells [46]. Hence, it is unsurprising that the role of NETs, which contain large amounts of proteases, have recently been investigated for their role in *Acanthamoeba* keratitis. *A. castellanii* trophozoites stimulate NET release, while their cysts fail to demonstrate such effects [48]. Furthermore, trophozoites appear to evade neutrophilic killing by NETs using their 3′-nucleotidase/nuclease capabilities [48]. To the best of our knowledge, this is the only report on the role of NETs in protozoal ocular diseases. Although protozoal ocular infections are rare, further investigations are warranted to investigate the NET-related mechanisms due to the critical sight-threatening potential of such diseases.

## 7. NETs in Non-Infectious Corneal Diseases

Dry eye disease (DED) affects millions of people across the globe and is the most common reason for visiting an ophthalmologist [49]. It presents variably, with patients having severe eye pain, low energy, and overall diminished health [49]. It is a multifactorial disorder characterized by tear film instability, ocular surface inflammation, and increased tear film osmolarity [16]. NETs have been seen in the tears of DED patients, suggesting their involvement in the pathogenesis of this condition [50]. The hallmark feature of DED is hyperosmolarity, which promotes the production of NETs [51]. Hyperosmolarity further exacerbates the condition by promoting the accumulation of NETs in the precorneal tear film [51]. Additionally, in conditions like meibomian gland dysfunction, the accumulation of NETs in the meibomian gland can lead to duct occlusion and acinar atrophy, further worsening the symptoms of DED [52].

Ocular graft-versus-host disease (GvHD) can also be an underlying cause of DED [53]. In murine models, NETs mediate several pathogenic effects by causing epitheliopathy, increasing T-cell proliferation, and inhibiting meibomian gland cell proliferation and differentiation [53]. NET-associated markers, such as oncostatin M and LIGHT/TNFSF14, are associated with these changes, revealing their potential as markers of severity or progression [53].

Mooren’s ulcer (MU) is a rare corneal disorder characterized by the presence of chronic, painful ulcers in the peripheral or central regions of the cornea, leading to stromal destruction [54]. The ulcer is initially present in the marginal area of the cornea and later progresses circumferentially, sometimes involving the entire cornea [55]. Although MU is classified as an autoimmune disease, the exact pathophysiology of MU is still not well understood [56].

There is growing evidence that dysregulated neutrophils and the formation of NETs play a significant role in the pathogenesis of MU. A study performed by Chi et al. aimed to investigate the underlying mechanisms of MU [54]. MU samples showed increased neutrophil infiltration compared with control samples. An increase in PAD4 and NET-related markers, such as AZU1, DEFA1, S100A8, ELANE, and MPO in MU samples was also noted. Furthermore, PAD4 levels were elevated [54].

Alkaline pH is also a known promoter of NET formation through Nox-independent mechanisms [57]. Hence, the role of NETs in alkali ocular burns is of interest. Wan et al. studied the impact of NET formation on alkali burns and how acetylsalicylic acid may modulate their formation [58]. First, their study demonstrated that alkali-activated neutrophils blunt the proliferation and migration of human corneal epithelial cells via phagocytosis and NET formation. Subsequently, acetylsalicylic acid was found to reverse these effects by inhibiting nuclear factor kappa-B (NF-κB) and, hence, reducing NET formation [58].

Other NET-targeting strategies have emerged for the treatment of DED. In fact, clinical trials have begun demonstrating the effectiveness of eye drops containing DNase, an enzyme responsible for NET degradation. For example, a phase I/II clinical trial revealed that DNase eye drops are safe and well-tolerated [59]. Additionally, a greater reduction in corneal staining and mucoid debris was seen in the intervention group compared with the placebo [59]. Sub-anticoagulant dose heparin has shown promising results in dismantling NETs and mitigating their detrimental effects by destabilizing NETs and preventing them from damaging epithelial and fibroblasts [53,60].

Recently, Nan et al. investigated the potential of bone morphogenetic protein (BMP)-4 as a therapeutic modality in corneal neovascularization [61]. Corneal neovascularization is a leading cause of blindness worldwide and results from an imbalance in pro- and anti-angiogenic factors in the cornea [62]. BMP4 was found to inhibit corneal neovascularization in vivo [61]. Among other effects, BMP4 inhibited NET generation through the Nox-dependent pathway. MPO is seen in increasing levels in models of alkali burn-related corneal neovascularization, which could be the potential mechanistic link between NETs and corneal neovascularization [63,64]. However, this has not been directly studied yet.

Although NETs are a significant aspect of corneal diseases, it is important to note that NETs are not the sole mediators of such diseases. NETs could provide valuable targets in the treatment of corneal disorders through either direct degradation or inhibition of NET pathways, such as NF-κB.

## 8. NETs in Dacryolithiasis

Dacryoliths, also known as mucopeptide concretions, are concretions that are in the lacrimal sac and duct, potentially leading to lacrimal obstruction [65]. Very little is known about the composition of dacryoliths and the mechanisms behind their formation. In a recent study, Zlatar et al. investigated these topics in the context of NETs [66]. First, the authors identified several antigens within dacryoliths, including NE, MPO, and PAD4 in 43%, 71%, and 100% of their samples, respectively. Then, the authors found extracellular DNA on the outer parts of the samples, characteristic of NETs. Their findings elucidated the role of NETs in dacryolithiasis in the chronic stages of the condition [66]. Nevertheless, there is still a large gap in the literature surrounding the pathogenesis of dacryolithiasis.

## 9. NETs in Non-Infectious Uveitis

Uveitis is a general term used to describe inflammation that affects the uveal tract of the eye, which consists of the choroid, ciliary body, and iris [67]. Inflammation can affect other nearby structures in the eye as well [67]. Uveitis is a sight-threatening condition that usually presents with eye pain, redness, blurred vision, and sensitivity to light [68]. Uveitis can be caused by a myriad of infectious and non-infectious etiologies [69]. The most common non-infectious causes include acute anterior uveitis (AAU), Bechet’s disease, Vogt–Koyangi–Harada, and juvenile idiopathic arthritis (JIA) [69]. Infectious uveitis is frequently caused by pathogens like herpes simplex virus, *Mycobacterium tuberculosis*, *Toxoplasma gondii*, and *Treponema pallidum* [70]. The pathogenesis of uveitis can be different depending on the condition and cause, but it is clear that NETs have a role in the progression and severity of the disease. As stated previously, NETs have been implicated in ERU and have potential prognostic utility [15]. In the following sections, we synthesize the evidence describing NETs in various forms of non-infectious uveitis.

### 9.1. Acute Anterior Uveitis

AAU is an ocular disease that involves inflammation of the anterior component of the eye, iris, and ciliary body. AAU is characterized by common symptoms of uveitis, lasting less than 3 months. AAU has been linked to the HLA-B27 gene, making it commonly found with other HLA-B27-associated diseases such as ankylosing spondylitis, Reiter’s syndrome, and other seronegative arthropathies [71].

The pathogenesis of AAU is poorly understood; however, different animal models have been used to demonstrate the role that cytokines might play [72,73]. These models describe the development of endotoxin-induced AAU following cytokine release by macrophages and the infiltration of neutrophils and mononuclear cells [73]. In the same study, it was revealed that the injection of endotoxins induces a massive influx of neutrophils into the anterior uvea [73]. Six weeks post-intervention, neutrophils can still be seen in the anterior uvea [73]. Neutrophils are naturally absent from the anterior uvea, indicating that neutrophils could be mediators of AAU [73]. The activation of resident macrophages leads to the release of inflammatory cytokines tumor necrosis factor (TNF)-ɑ and IL-1 [73]. As the inflammatory cascade potentiates and more cytokines are released, neutrophils and different inflammatory cells are attracted into the anterior uvea [73]. While some inflammatory cells die early, neutrophils live on in the anterior uvea and maintain tissue damage through the production of different mediators including TNF- and nitric oxide [73]. However, studies have yet to determine the presence and role of NETs in the pathogenesis of the disease. Further research is needed in this regard.

### 9.2. Behcet’s Disease

Behcet’s Disease (BD) is a systemic vasculitis that causes repeated acute attacks of inflammation in vascular areas of the body [74]. Although the pathogenesis of BD remains unclear, it has been suggested that immunological factors play a role in the development of the disease in genetically predisposed individuals, such as those with HLA-B51 [74,75]. BD manifestations include major vessel disease, eye disease, and central nervous system involvement [74]. Uveitis is the most common ocular manifestation of BD and may even lead to blindness [76].

Following exposure to environmental antigens, the innate immune response is activated, leading to the activation and migration of T-cells, natural killer cells, and neutrophils [74]. Neutrophils play an important role in innate immunity; however, they are notorious for causing exacerbations by damaging host cells [74]. Neutrophils promote the pathogenesis of BD by producing ROS and NETs [74,77]. BD patients have been shown to exhibit more NETosis compared with healthy individuals [78]. NETs cause host tissue damage through several mechanisms [77,78]. First, NETs are internalized and degraded by macrophages, causing a feed-forward loop of cytokine release and inflammatory cell recruitment [74,78]. NETs have been shown to promote pro-coagulant states in animal models [77], a characteristic feature of BD [79]. BD-specific mechanisms related to NET release were unstudied until recently. Recently, it was proven that IL-8 promotes NET formation through Nox-dependent and Nox-independent pathways [80]. Additionally, IL-8-triggered NETs directly surrounded the studied cells [80]. IL-8 is an inflammatory marker used to monitor BD activity, with some reporting it to be a more reliable marker than the frequently used C-reactive protein and erythrocyte sedimentation rate [81,82]. Treatment with SB225002, a potent antagonist of the relevant pathways, significantly blunted the release of NETs in animal models [80].

Further reinforcing the role of neutrophils and NETs in BD, colchicine, an anti-neutrophilic drug, significantly reduces NET production and cell apoptosis in vitro [75]. In line with this, colchicine has shown some promising outcomes in BD patients [83]; however, this has not yet been studied in large-scale studies, to the best of our knowledge. Further research is needed to understand the full picture of the pathogenesis of BD and the implications of counteracting neutrophils as possible management.

### 9.3. Juvenile Idiopathic Arthritis

JIA is a chronic rheumatic disease of unknown etiology that presents in children [84]. Uveitis is among the most severe extra-articular manifestations of JIA and can lead to serious complications [84]. The cause of ocular involvement is not fully understood, but there have been reports of a strong association between JIA and uveitis for many years [85]. In genetically susceptible populations, certain triggers can cause the development of JIA via the release of pathogen- and damage-associated molecular patterns that jump-start the inflammatory process [86]. These inflammatory triggers lead to macrophage activation and neutrophil transmigration into affected tissues [86].

Neutrophil activation is an important factor in the pathogenesis of JIA and serves as a differentiating point from other rheumatic diseases [86]. Following neutrophil arrival at the site of inflammation, they are responsible for the expression of various chemokines including CXCL-9 to 13 and CXC receptor 4 [86]. The activation and migration of neutrophils are further exacerbated by the cytokine release from macrophages [86]. Recent studies have even suggested that NETs play a significant role in potentiating JIA-related inflammation [86]. The serum levels of NET-related markers are positively associated with JIA severity and are markedly elevated in JIA patients compared with healthy controls [14,87]. NET-releasing neutrophils are significantly more prevalent in patients with active JIA compared with those with the remitting form [87]. The pathogenic effects of NETs are believed to stem from the activation of TLR4 on macrophages [86]. TLR4 then functions via the NF-κB pathway to increase the expression of pro-inflammatory cytokines, promoting even further neutrophil activation and recruitment [86]. In terms of therapeutic value, heparin reduces neutrophil activation and, accordingly, NET release in systemic-onset JIA [87]. More research is needed in this area to understand the pathogenesis of JIA and what drives neutrophils to be hyperactive in these individuals and foster disease progression.

## 10. NETs in Diabetic Retinopathy

Diabetic retinopathy (DR), is a common complication affecting the microvasculature in patients with diabetes, affecting nearly one-third of patients [88]. Chronically elevated blood sugar results in inflammatory and oxidative cellular damage, breaching the integrity of the inner blood–retina barrier. As a result, plasma leaks out to retinal cells, causing retinal cell injury and the release of cytokines [89,90].

In addition to the pro-inflammatory state seen in diabetic patients, hyperglycemia itself is a trigger for NET release [18]. An increased concentration of circulating NET biomarkers, such as DNA-histone complexes and polymorphonuclear NE, has been associated with an increased risk of DR [91]. Song et al. confirmed that elevated blood glucose increases the levels of DNA-histone complexes and activates factor XII, which increases the risk of DR [92]. The presence of NETs is also significantly associated with DR compared with non-DR patients [93]. Additionally, NETs have recently been associated with elevated levels of glucose and glycated hemoglobin and a poor estimated glomerular filtration rate, indicating its potential in monitoring and, potentially, predicting diabetes progression [93]. Various authors have discovered increased levels of NET components (e.g., eDNA, NE, PO, LCN2) in the retina of diabetic rats and in the blood and vitreous fluid of DR patients [94]. On the other hand, a study by Binet et al. showed a beneficial effect of NETosis. NETs aided in the elimination of senescent endothelial cells, resulting in the regression of pathological angiogenesis and the regeneration of functional vessels [95].

NETs may also prove valuable in the management of DR. Barliya et al. demonstrated that DNase treatment significantly reduces NET levels in the anterior and posterior chambers of proliferative DR mice [96]. Whether these findings translate into the clinical setting has yet to be studied, however.

## 11. Age-Related Macular Degeneration

Age-related macular degeneration (AMD) is the third leading cause of blindness worldwide [97]. AMD is associated with drusen deposition, which causes a disturbance in the metabolic connection between the retinal pigment epithelium (RPE) and choroid. This ultimately leads to choroidal neovascularization during the late stages of AMD [98]. Abnormal levels of citrullinated proteins have been found in AMD retinas, despite similar levels of PAD2 enzymes in both AMD and non-AMD postmortem patients [99]. In a meta-analysis by Niazi et al., the neutrophil-to-lymphocyte ratio (NLR) was significantly greater in AMD patients [100]. However, when further stratified, the difference was limited to neovascular AMD, and no difference was seen between dry AMD patients and controls.

Significant infiltrations of lipocalin-2 (LCN2)-secreting neutrophils can be seen in AMD retinas, and these neutrophils have been shown to overexpress matrix metallopeptidase (MMP)-9 enzymes. The interaction between LCN2 and MMP-9 appears to form a complex in the outer retina–choroid region of AMD patients. As a result, the LCN2/MMP-9 complex can potentially cause choroidal vascularization in AMD, similar to what occurs in corneal vascularization [101,102]. LCN2, a component of NETs, is a neuroinflammatory protein responsible for promoting neutrophil migration into the retina, where, in conjunction with other retinal cells, they synthesize and release new LCN2, leading to further potentiation of retinal damage [102]. The authors of this discovery also reported neutrophil expression of other NET proteins such as MPO and NE [102]. Specifically, A*β*1-40, the main component of drusen, triggers NET formation through TLR4- and Nox-mediated pathways, in AMD mice models [96]. The inhibition of NETs in these models alleviates retinal inflammation, further demonstrating the pathogenic role of NETs in AMD [96]. In addition, neutrophils play a role in secreting pro-angiogenic factors and inflammatory cytokines like vascular endothelial growth factor and IL-8, leading to the recruitment of other angiogenic cells and acting as a platform for choroidal neovascularization [97,103]. Although there is reported evidence of NETs in AMD retinas, explanations of how NETs impact AMD remain limited.

## 12. Conclusions

**Figure 2:**
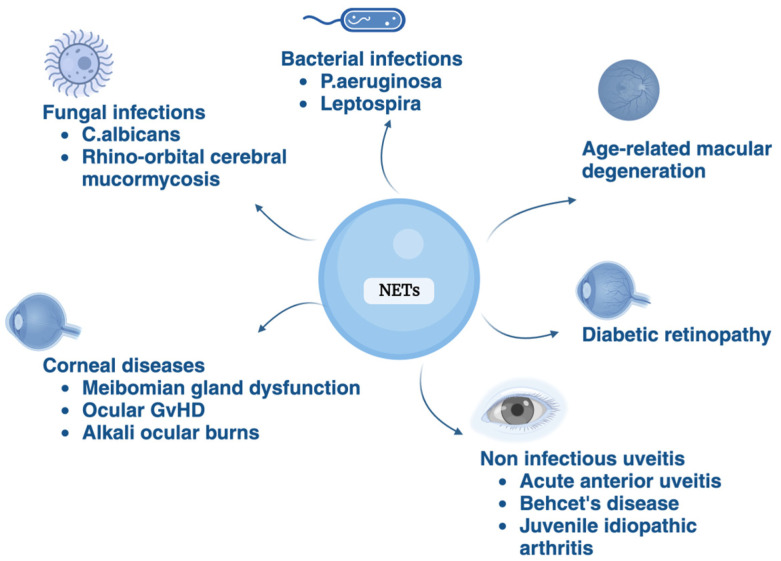
NETs play several pathological roles in the eye.

NETs manifest a dichotomy of function, serving both protective and pathogenic roles in ocular diseases. While NETs exhibit a pivotal protective role by safeguarding the eye from debris and modulating proinflammatory cytokines under normal physiological conditions, their involvement in various ocular diseases, such as corneal diseases, uveitis, diabetic retinopathy, and AMD, highlights a complex pathogenic potential (Figure 2). The molecular mechanisms underlying NET formation and their nuanced roles in ocular health and disease present a fertile ground for future research.

Targeting NET pathways, despite showing promising results, necessitates further comprehensive studies to unravel their full potential and limitations in treating ocular conditions. Future research should strive to delineate the intricate balance between the beneficial and detrimental effects of NETs in the eye, aiming to harness their protective attributes while mitigating their pathogenic impacts. In doing so, it is imperative to conduct extensive clinical trials to ascertain the safety, efficacy, and applicability of NET-targeting therapies in diverse patient populations and ocular diseases.

This multifaceted approach will not only deepen our understanding of ocular pathophysiology but also potentially open new horizons for innovative, targeted, and effective therapeutic interventions in ocular medicine. It is with anticipation and optimism that we await further research endeavors exploring the enigmatic world of NETs, hoping to illuminate the dark corners of ocular disease pathogenesis and therapy.

## Data Availability

No original data were generated in this study.
